# The Future of Serum-Therapy

**Published:** 1897-09

**Authors:** 


### THE FUTURE OF SERUM-THERAPY.

THE serum-treatment of diphtheria in Berlin is said to have brought the average mortality down to 12.3 per cent. in a total of 5794 cases. Its application to tuberculosis goes on, and gratifying results are being reported. Dr. Paul Paquin reports some 226 cases treated with antitubercle-serum, with forty recoveries and a large number of improvements. Leprosy, cancer, Asiatic cholera, swine-plague, and snake-bite are all new fields for the employment of the serum-treatment, and the future is full of promise in these disastrous and painful afflictions. In comparative medical science we have many promising fields for the employment of treatment along these lines, and with many new workers in the field we are sure that much will come from their labors. The losses of a monetary character in the live-stock industry are such as to demand more efficient measures and to warrant the most extended investigations, with the hope that the losses may be in a great measure controlled. We are glad to note that the General Government, and several of our State governments, notably Nebraska, Pennsylvania, Minnesota, Wisconsin, and Texas are making appropriations of money, and are securing the best talent to work out these perplexing problems.

